# High‐Sensitive Cardiac Troponin I and Th1/Th2 Polarization in Queens With Pyometra

**DOI:** 10.1002/vms3.70125

**Published:** 2025-01-10

**Authors:** Tarik Safak, Nevzat Saat, Oznur Yilmaz‐Koc, Mert Turanli, Aslıhan Ayalp‐Erkan, Ali Risvanli

**Affiliations:** ^1^ Faculty of Veterinary Medicine Department of Obstetrics and Gynecology Kastamonu University Kastamonu Turkey; ^2^ Faculty of Veterinary Medicine Department of Obstetrics and Gynecology Balikesir University Balikesir Turkey; ^3^ Faculty of Veterinary Medicine Department of Obstetrics and Gynecology Siirt University Siirt Turkey; ^4^ Faculty of Veterinary Medicine Department of Obstetrics and Gynecology Firat University Elazig Turkey; ^5^ Faculty of Veterinary Medicine Department of Obstetrics and Gynecology Kyrgyz‐Turkish Manas University Bishkek Kyrgyzstan

**Keywords:** high‐sensitive cardiac troponin I, pyometra, queen, Th1/Th2 cytokine polarization

## Abstract

**Objectives:**

To determine T helper (Th)1 and Th2 cytokine polarization, as well as high‐sensitive cardiac troponin I (hs‐cTnI) levels, in cats with pyometra.

**Methods:**

We used 40 queens in the study. A total of 20 out of these 40 queens were diagnosed with the pyometra group (PYO) and the other 20 made up the healthy group (control; CTR). We measured concentrations of hs‐cTnI, aspartate aminotransferase (AST), creatine kinase (CK) and l‐lactate in queens from both groups. Additionally, we measured cytokine concentrations in all queens.

**Results:**

The hs‐cTnI concentration in the PYO group (26.95 ± 5.08 ng/L) was significantly higher than that of the CTR group (7.00 ± 0.82 ng/L) (*p* < 0.000). Furthermore, the PYO group had a higher CK concentration (344.50 ± 39.63 U/L) than the CTR group (191.00 ± 15.44 U/L) (*p* = 0.002). The PYO group also demonstrated higher concentrations of TNF‐*α* (9.77 ± 0.81 ng/mL), IFN‐*γ* (25.37 ± 2.09 ng/mL), IL‐2 (4.37 ± 0.39 ng/mL), IL‐4 (245.64 ± 15.83 pg/mL), IL‐5 (63.13 ± 1.65 pg/mL) and IL‐10 (123.58 ± 4.30 ng/mL) compared to the CTR group (*p* < 0.000).

**Conclusions:**

Overall, it is suggested that changes in cytokine concentrations increase in queens with pyometra, potentially causing harm to the heart muscle. It is crucial to consider that the heart muscle may also be affected in queens with pyometra during the treatment process.

## Graphical abstract

• This study investigates the relationship between hs‐cTnI and Th1/Th2 cytokines polarization in queens suffering from pyometra.

• A correlation was found between hs‐cTnI and CK concentrations and cytokines in queens with pyometra.

• hs‐cTnI is extremely important in developing treatment methods against pyometra, protective measures and monitoring heart muscle damage in cats with pyometra.

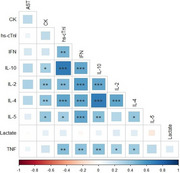

## 1 Introduction

Pyometra is a reproductive disease caused by bacteria and hormones, occurring during the luteal phase in intact queens. This condition is characterized by the accumulation of pus in the uterine lumen (Hagman 2018). The incidence of pyometra is higher in the diestrus phase due largely to increased progesterone levels. The use of external hormones, like progesterone for estrus suppression, may also contribute to this condition (Keskin et al. 2009). High progesterone concentrations enhance the risk of pyometra through immune system suppression. Although the etiopathology of pyometra has been researched for many years, it is still not fully understood. Impaired immune function, including reduced leukocyte activity, is associated with this disease (Bartoskova et al. 2012; Hollinshead and Krekeler 2016). Bacteria such as *Escherichia coli* can proliferate in the uterine lumen, with potential complications of pyometra induced by *E. coli* including fatal sepsis and endotoxemia. The endotoxin released by *E. coli* is a highly potent inflammatory stimulant (Xavier et al. 2022). The mucosal membranes, including the endometrium, play an essential role in fending off pathogens. Cells within the mucosa, whether epithelial or stromal, employ pattern‐recognition receptors to detect pathogens. These receptors identify pathogen‐associated molecular patterns (PAMPs) (Kakihana et al. 2016). PAMPs can include microbial components like lipopolysaccharide (LPS), lipoteichoic acid and lipoproteins. Toll‐like receptors (TLRs) are involved in recognizing these PAMPs. When TLRs are activated, they influence the secretion of cytokines like interleukin‐1 (IL‐1), IL‐2, IL‐6, IL‐8, IL‐10 and tumour necrosis factor‐alpha (TNF‐*α*) (Jursza et al. 2015, 2014; Jaffer, Wade, and Gourlay 2010).

Cytokines produced by Th1 cells include interferon (IFN)‐*γ*, IL‐2, TNF‐*α* and IL‐1β, whereas Th2 cells produce cytokines such as IL‐4, IL‐5, IL‐10 and IL‐13. Th1 cells release cytokines that promote the cellular immune response, while cytokines released by Th2 cells activate humoral immunity (Zhu and Paul 2008; Dembic 2015).

Besides immune changes, conditions like pyometra also affect organs such as the liver, kidney and heart in cats, leading to alterations in organ‐specific biochemical parameters in the bloodstream (Singh et al. 2020; dos Santos Silva et al., 2024). Studies in dogs and cats diagnosed with pyometra have identified changes in biochemical parameters including alanine aminotransferase (ALT), albumin (ALB), gamma‐glutamyltransferase (GGT), alkaline phosphatase (ALP), aspartate aminotransferase (AST), creatine kinase (CK), blood urea nitrogen (BUN) and l‐lactate (Maddens et al. 2011; Vijay et al. 2021; Satilmis 2023; Friolani et al. 2024). Moreover, elevated creatinine and high‐sensitive cardiac troponin I (hs‐cTnI) levels indicate heart muscle damage (Langhorn and Willesen 2016; Wesselowski et al. 2023). Serum creatinine can function as a prognostic marker for canine pyometra, considering its association with sepsis (Sant'Anna et al. 2014). Research has been conducted indicating that cTnI could be used as the most accurate and responsive marker for different diseases in different animal species (Labonté et al. 2018; Leonardi et al. 2008; Tümer, Çalişkan, and Şafak 2021; Tümer and Safak 2022). In addition, this marker has been investigated in dogs with pyometra (Hagman et al. 2007; Pelander, Hagman, and Häggström 2008). However, not many studies have been conducted on queens with pyometra. Many queens with pyometra recover successfully after surgery, though preoperative, intraoperative and postoperative complications may occur (Dąbrowski et al. 2009). Furthermore, the biochemical changes observed in queens with pyometra infection are typically less distinct than those found in bitches (Satilmis 2023).

There is little available information on T helper (Th)1/Th2 cytokine polarization and the changes in hs‐cTnI, AST, CK and l‐lactate concentrations in queens with pyometra. Therefore, the primary aim of this study is to assess the serum concentrations of hs‐cTnI, AST, CK and l‐lactate, as well as Th1/Th2 cytokine polarization, TNF‐*α*, IFN‐*γ*, IL‐2 / IL‐4, IL‐5, IL‐10, respectively.

## 2 Materials and Methods

### 2.1 Ethical Approvals

The study received ethics committee approval from the Balıkesir University Animal Experiments Local Ethics Committee (2024/2‐5). The owners of the queens signed an informed consent form before evaluation and treatment.

### 2.2 Animals

The materials for the research study comprised queens delivered to Balıkesir University's Faculty of Veterinary Medicine, Department of Obstetrics and Gynecology, for examination. We utilized 40 mixed‐breed queens aged between 1 and 7 years (mean age 4.93 ± 2.9) and weighing between 2.2 and 4.5 kg (average weight 2.9 ± 0.6) in this study. We also reported the presence or absence of clinical signs of systemic illnesses such as depression, loss of appetite, fever, polyuria, polydipsia, vomiting and diarrhoea.

### 2.3 Abdominal Ultrasonography and Radiography of Genital Organs and Vaginal Smears

All queens underwent abdominal ultrasonography and radiography. The radiological examination incorporated left latero‐lateral and ventral‐dorsal projections of the entire abdomen using a standard procedure (Hayati et al. 2016).

Ultrasonography was performed in the ventro‐dorsal position as described by Zambelli et al. (2006). The ovaries and uterus were visualized during this examination. We used a B‐mode ultrasonography with a 5–7.5 MHz transducer and a convex probe (Versana Active, USA) for the transabdominal ultrasound test to determine if the queens had pyometra. Queens diagnosed with pyometra were included in the study, forming the pyometra group (PYO, *n* = 20).

A vaginal smear was further performed to determine the sexual cycles of queens, excluding those not pregnant. The smears and consequent analysis of sexual cycles were carried out as outlined by Termelioğlu, Kalender, and Erat (2022). For the smear, cells from the vaginal tissue were collected using a cotton swab and spread onto a clean, pre‐labelled slide. The collected cells were air‐dried and affixed with ethyl alcohol on the slide. Subsequently, the slides were completely covered with Giemsa stain using a dropper, ensuring an even spread across all regions. Following air‐drying, the stained vaginal smear preparations were assessed under a light microscope (Leica DM500, Germany). Cells were classified based on their morphology as parabasal, intermediate, surface or keratinized superficial. Queens in an anoestrus state, according to cell profiles, were assigned to a control group (CTR, *n* = 20). Pregnant queens were not included in this study.

### 2.4 Blood Samples and Haematological Analysis

Approximately 3 mL of blood was collected from each group of queens and transferred to a gel serum tube (BD, Plymouth, UK) containing a clot activator. Blood samples for serum hs‐cTnI, cytokine and biochemical analyses were centrifuged at 20 g for 10 min, after which the sera were separated and stored at −20°C until analysed. Additionally, blood samples were collected into 0.5 mL tubes (BD, Plymouth, UK) containing tripotassium ethylenediaminetetraacetic acid (K3EDTA). In the haematological analysis, variables, including white blood cells (WBCs), red blood cells (RBCs), haematocrit (HCT), haemoglobin (HGB) and platelet count (PLT) were measured using an automatic haematology instrument (Norma Ivet‐5, Hungary).

### 2.5 High‐Sensitive Cardiac Troponin I and Biochemical Analysis

After thawing, the serum concentrations of hs‐cTnI in 40 queens were evaluated using a chemiluminescent immunoassay designed for the detection of human‐based hs‐cTnI (ADVIA Centaur XP High‐Sensitive cTn‐I, Siemens Healthcare Diagnostics). According to the manufacturer, the measurement range of this assay is 2.5–25,000 ng/L. This old‐generation human‐based device (ADVIA Centaur CP TnI‐Ultra) has been used and validated in cats in previous studies (Langhorn, Willesen, et al. 2013). The new‐generation ADVIA hs‐cTnI device (ADVIA Centaur XP) has been validated in dogs (Wesselowski et al. 2023). The concentrations of AST, CK and l‐lactate in serum samples were measured with an automatic biochemistry analyser (Diasys Respons 910Vet, Holzheim, Germany).

### 2.6 Cytokine Analysis

Cytokine concentrations were measured using the enzyme‐linked immunosorbent assay (ELISA) test method with commercial TNF‐*α* (Cat no: E0031Cat, Bioassay Technology Laboratory, Shanghai, China), IL‐4 (Cat no: E0121Cat, Bioassay Technology Laboratory), IL‐5 (Cat no: E0122Cat, Bioassay Technology Laboratory), IL‐2 (Cat no: E0051Cat, Bioassay Technology Laboratory), IFN‐*γ* (Cat no: E0013Cat, Bioassay Technology Laboratory) and IL‐10 (Cat no: E0030Cat, Bioassay Technology Laboratory). The stages of the ELISA test process were conducted according to the manufacturer's instructions and the methods detailed in the literature (Safak, Risvanli, and Asci‐Toraman 2022). Upon completion of all processes, serum cytokine concentrations were determined by reading the plates at 450 nm in an ELISA reader.

### 2.7 Statistical Analysis

The statistical analysis was conducted using SPSS 22 software (SPSS version 22.0 for Windows; SPSS Inc., Chicago, USA). The normal distribution presence in the data was assessed using the Kolmogorov–Smirnov test. The Mann–Whitney *U* test was employed to analyse the statistical differences between groups. *p*‐values were set at 0.05. The association between the variables was analysed using Spearman's rank correlation test. The correlation coefficients’ values were interpreted as follows: *r* = 0.00–0.10 (negligible correlation), *r* = 0.10–0.39 (weak correlation), *r* = 0.40–0.69 (moderate correlation), *r* = 0.70–0.89 (strong correlation), and *r* = 0.90–1.00 (very strong correlation) (Schober, Boer, and Schwarte 2018).

## 3 Results

The WBC value in the PYO group (27.55 ± 2.37 × 10^3^/µL) was found to be higher than that in the CTR group (10.20 ± 0.70 × 10^3^/µL; *p* < 0.000). On the other hand, RBC (8.24 ± 0.37 × 10^6^/µL), PLT (300.00 ± 40.22 × 10^3^/µL; *p* < 0.05), HGB (13.10 ± 0.67 g/dL) and HTC (45.00 ± 2.35%; *p* < 0.000) values were observed to be higher in the CTR group (Table 1).

**TABLE 1:** Haematological analysis of queens in the pyometra (PYO) and control (CTR) groups (median ± standard error of the mean).

	Parameters				
Groups	WBC (×10^3^/µL)	RBC (×10^6^/µL)	HGB (g/dL)	HCT (%)	PLT (×10^3^/µL)
CTR (*n* = 20)	10.20 ± 0.70	8.24 ± 0.37	13.10 ± 0.67	45.00 ± 2.35	300.00 ± 40.22
PYO (*n* = 20)	27.55 ± 2.37	7.38 ± 0.23	10.20 ± 0.25	31.0 ± 1.60	204.00 ± 23.43
P	0.000	0.014	0.000	0.000	0.020

Abbreviations: CTR, control group; HCT, haematocrit; HGB, haemoglobin; PLT, platelet count; PYO, pyometra group; RBC, red blood cell; WBC, white blood cell.

**FIGURE 1:**
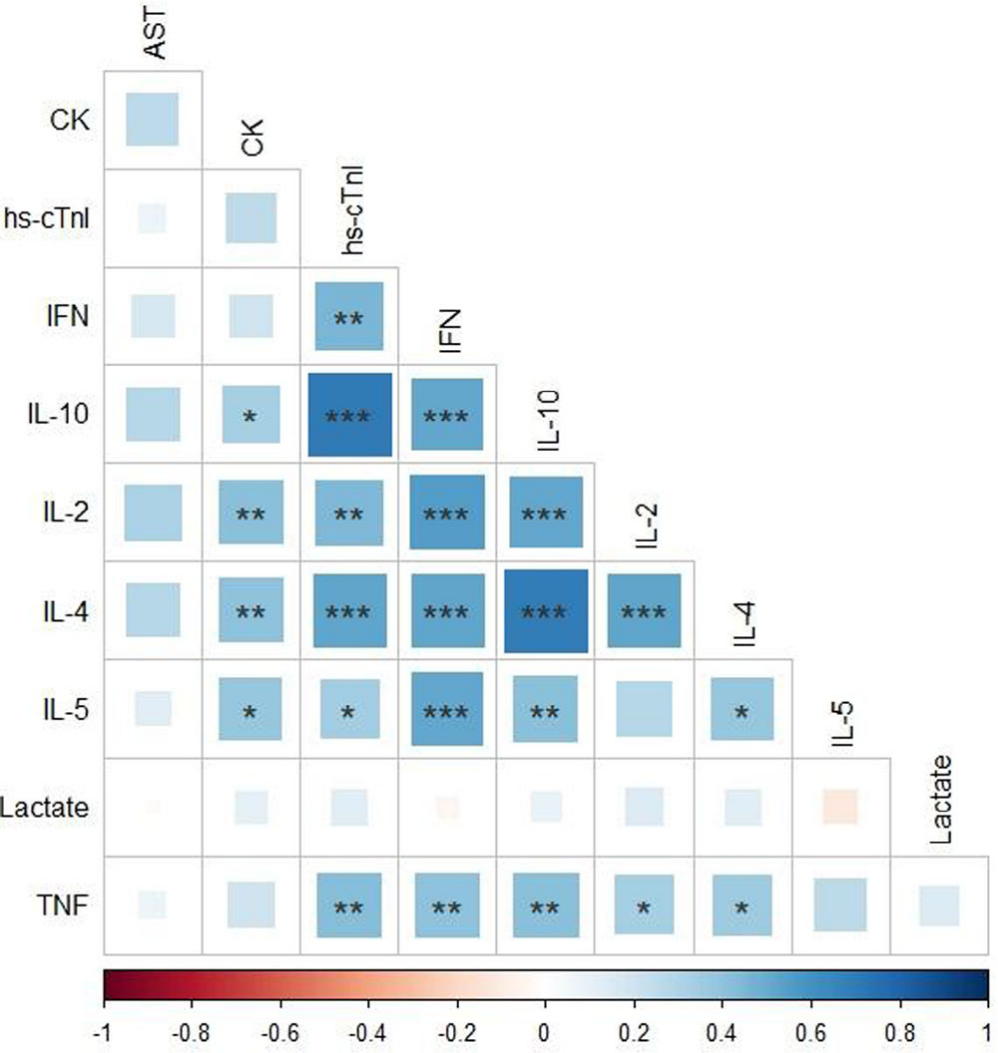
The relationship between the parameters was made by Spearman's correlation analysis. Positive correlations are shown in blue and negative correlations in red as shown in scale gradient at the bottom of the graph. The colour intensity of the boxes is proportional to the correlation coefficients. Only significant correlations are indicated by an asterisk inside the box; *p < 0.05; **p < 0.01; ***p < 0.001.

Although hs‐cTnI shows a strong correlation with both IL‐10 and IL‐4 (*p* < 0.001), it also has a strong correlation with IFN‐*γ*, IL‐2 and TNF‐*α* (*p* < 0.01). The results indicate a moderate correlation between IL‐5 and hs‐cTnI (*p* < 0.05). There is a strong correlation between CK with both IL‐2 and IL‐4 (*p* < 0.01) and a moderate correlation between CK, IL‐10 and IL‐5 (*p* < 0.05; Figure 1).

**FIGURE 2:**
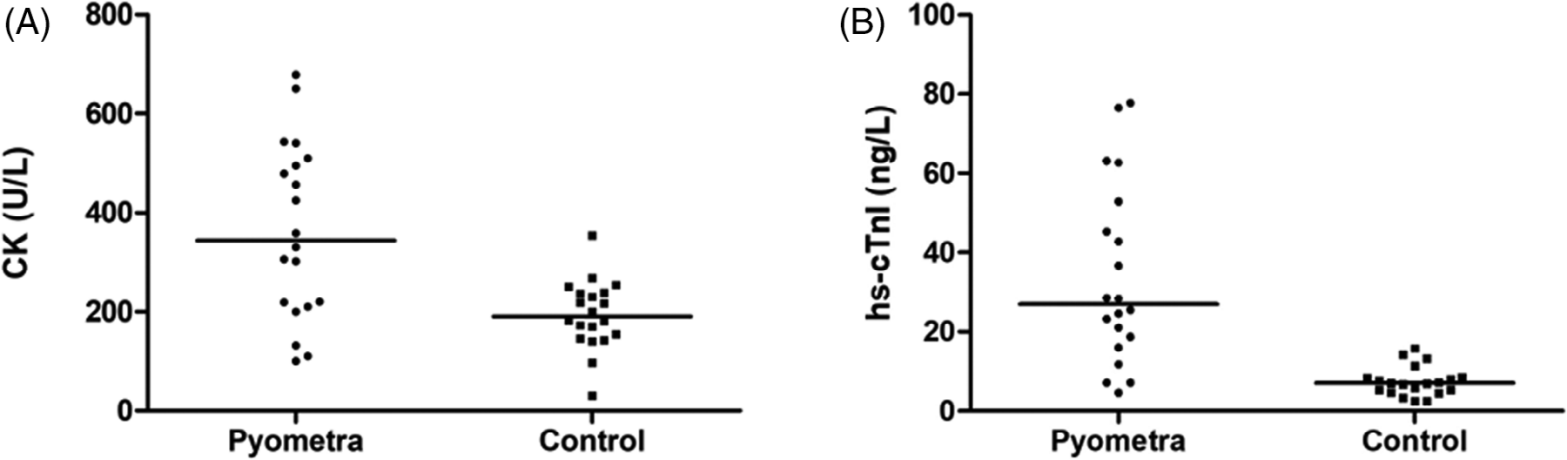
Serum creatine kinase (CK) (A) and high‐sensitive cardiac troponin I (B) concentrations of PYO and CTR groups. (*p* = 0.002 and *p* = 0.000, respectively.)

The hs‐cTnI concentration in the PYO group (26.95 ± 5.08 ng/L) was found to be significantly higher compared to the CTR group (7.00 ± 0.82 ng/L; *p* < 0.000; Figure 2B). Moreover, the PYO group exhibited a higher CK concentration (344.50 ± 39.63 U/L) than the CTR group (191.00 ± 15.44 U/L; *p* = 0.002; Figure 2A). However, there was no statistically significant difference found in the concentrations of AST and l‐lactate (*p* = 0.081 and *p* = 0.355, respectively; Table 2).

**TABLE 2:** Serum biochemical analysis in the pyometra (PYO) and control (CTR) groups (median ± standard error of the mean).

	Parameters			
Groups	hs‐cTnI (ng/L)	AST (U/L)	CK (U/L)	l‐Lactate (mmol/L)
PYO (*n* = 20)	26.95 ± 5.08	45.50 ± 3.49	344.50 ± 39.63	4.13 ± 0.51
CTR (*n* = 20)	7.00 ± 0.82	34.00 ± 3.93	191.00 ±15.44	3.63 ± 0.41
*p*	0.000	0.081	0.002	0.355

Abbreviations: AST, aspartate aminotransferase; CK, creatine kinase; CTR, control group; hs‐cTnI, high‐sensitive cardiac troponin I; PYO, pyometra group.

**FIGURE 3:**
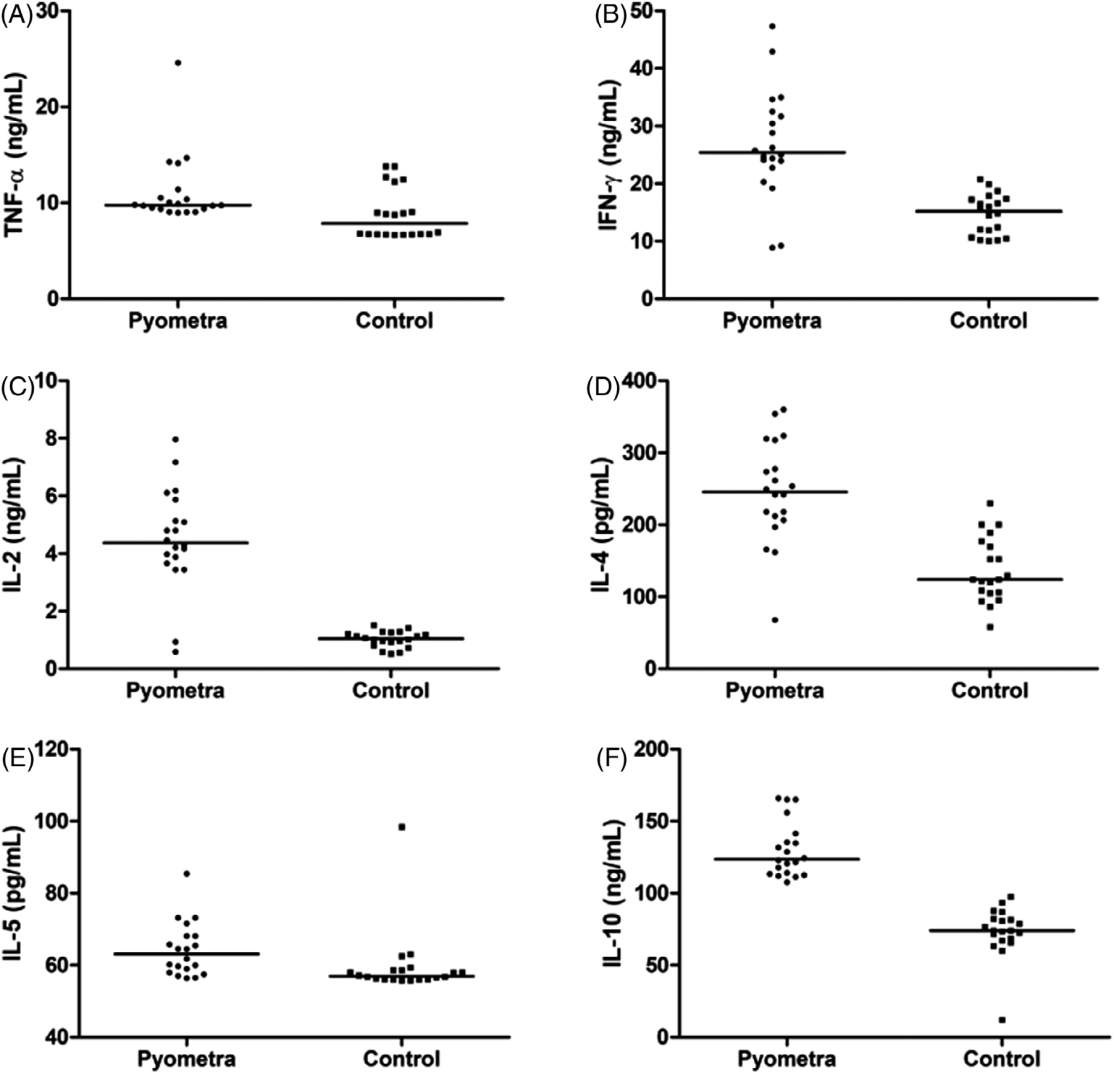
Serum tumour necrosis factor‐alpha (TNF‐*α*) (A), interferon‐gamma (IFN‐*γ*) (B), interleukin‐2 (IL‐2) (C), IL‐4 (D), IL‐5 (E) and IL‐10 (F) of PYO and CTR groups. *p*‐values are *p* < 0.01.

In the PYO group, elevated concentrations of TNF‐*α* (9.77 ± 0.81 ng/mL), IFN‐*γ* (25.37 ± 2.09 ng/mL), IL‐2 (4.37 ± 0.39 ng/mL), IL‐4 (245.64 ± 15.83 pg/mL), IL‐5 (63.13 ± 1.65 pg/mL) and IL‐10 (123.58 ± 4.30 ng/mL) were observed compared to the CTR group (Figure 3). The *p*‐values are *p* < 0.01 (Table 3).

**TABLE 3:** Cytokine concentrations in the pyometra (PYO) and control (CTR) groups (median ± standard error of the mean).

	Cytokines					
Groups	TNF‐*α* (ng/mL)	IFN‐*γ* (ng/mL)	IL‐2 (ng/mL)	IL‐4 (pg/mL)	IL‐5 (pg/mL)	IL‐10 (ng/mL)
PYO (*n* = 20)	9.77 ± 0.81	25.37 ± 2.09	4.37 ± 0.39	245.64 ± 15.83	63.13 ± 1.65	123.58 ± 4.30
CTR (*n* = 20)	7.86 ± 0.58	15.20 ± 0.77	1.04 ± 0.06	123.80 ± 10.08	56.95 ± 2.09	74.02 ± 3.90
*p*	0.001	0.000	0.000	0.000	0.001	0.000

Abbreviations: CTR, control group; IFN‐*γ*, interferon‐gamma; IL, interleukin; PYO, pyometra group; TNF‐*α*, tumour necrosis factor‐alpha.

## 4 Discussion

Cardiac troponins are recognized as a highly reliable indicator for identifying myocardial injury in both humans (Wu and Jaffe 2008) and animals (Langhorn and Willesen 2016), particularly in diseases that lead to cardiac damage. Conversely, elevated levels of cTnI have been observed in both cats (Hori et al. 2018) and dogs (Oyama and Sisson 2004), irrespective of the presence or absence of systemic cardiac disease. Earlier research discovered elevated circulating cTnI concentrations in naturally occurring sepsis (Kırbaş et al. 2021) or experimental sepsis models (Tümer, Özdemir, and Eröksüz 2020) in animals. Septicaemia occurs in cats and dogs that develop pyometra (Hagman 2018). In addition, organs such as the liver and kidneys are influenced by sepsis in animals (Brady and Otto 2001). Renal function often becomes compromised, potentially leading to secondary uremic myocardial damage in dogs with pyometra (Maddens et al. 2011). Similar to these organs, the heart muscle experiences damage caused by septicaemia. A study in dogs revealed cTnI as an indicator of heart muscle damage. High levels of endotoxins in the plasma were observed in bitches with pyometra. This endotoxin presence in the blood could be one reason for the increased levels of cTnI (Hagman et al. 2007).

In this study, the serum hs‐cTnI concentration in cats developing pyometra was found to be higher than in healthy cats. According to existing literature, the hs‐cTnI concentration has not been thoroughly investigated in cats with pyometra. However, cTnI concentration in dogs with pyometra was found to be higher than in healthy dogs. These findings are consistent with previous studies conducted in dogs (Hagman et al. 2007; Pelander, Hagman, and Häggström 2008).

The degree to which endotoxins are released can cause varying degrees of inflammation, from local to systemic, as well as cellular damage that may affect cardiac cells and elevate cTnI concentrations in serum (Pugliese et al. 2023). Endotoxins trigger inflammation and cytokine release by binding to receptors on cell membranes (Karabulut and Sönmez 2018). Cytokines are molecules that influence immune cell functions and the trajectory of the immune response, and they are often used in immune system evaluations. Th1 cells secrete cytokines such as TNF‐*α*, IFN‐*γ* and IL‐2, whereas Th2 cells secrete IL‐4, IL‐5 and IL‐10 (Heilmann and Suchodolski 2015).

In this study, the concentrations of TNF‐*α*, IFN‐*γ*, IL‐2, IL‐4, IL‐5 and IL‐10 in the PYO group were found to be higher than in the CTR group, at 9.77 ± 0.81 ng/mL, 25.37 ± 2.09 ng/mL, 4.37 ±0.39 ng/mL, 245.64 ± 15.83 pg/mL, 63.13 ± 1.65 pg/mL and 123.58 ± 4.30 ng/mL, respectively. According to Maciel et al. (2014), TNF‐*α*, IFN‐*γ*, IL‐2, IL‐4 and IL‐10 cytokine concentrations were found to be high in dogs with pyometra. TNF‐*α* is an early indicator of endotoxin exposure in animals with sepsis (Otto, 2007). However, in another study, no increase in these cytokines (TNF‐*α* and IL‐10) was observed in dogs with pyometra. In the same research, a statistical difference was detected between IL‐7 and IL‐8 (Karlsson et al. 2012). Finally, in the study by Karabulut and Sönmez (2018), they stated that monitoring circulating TNF‐*α* and IL‐6 concentrations is highly useful for scientists and clinicians in tracking inflammation in animals after ovariohysterectomy, especially in dogs with pyometra.

IL‐4 has been identified as an early indicator of mortality in severe cases of sepsis in humans (Bozza et al. 2007). This finding is also supported by studies conducted in dogs. Hence, it has been reported that elevated IL‐4 levels might serve as an early indicator of pyometra and determine the severity of the disease in female dogs (Maciel et al. 2014). Consistent with the research conducted by Maciel et al. (2014), in our investigation, IL‐4 concentrations could be used to measure the severity of pyometra in cats. However, as the onset of the disease was not precisely determined in our study, this hypothesis could be strengthened by experimental pyometra studies.

In a study conducted by Maciel et al. (2014), concentrations of IL‐10, IL‐4, TNF‐*α* and IFN‐*γ* were shown to be similar during both the pyometra and diestrus periods. This might be attributed to the immunosuppressive properties of progesterone, with a high concentration in a dog's circulation during pyometra and diestrus. In our study, all of the cats in the control group were in the anoestrus phase. Therefore, progesterone levels are typically low during a cat's anoestrus phase. Thus, significant differences were observed in these cytokine concentrations between the pyometra and the control groups. Even if there was a difference among cats in the oestrous phase, it is unlikely that such a disparity would have occurred.

Cytokines are thought to have a significant impact on causing myocardial damage in inflammation‐related diseases. Troponin concentrations in myocardial patients have been found to correlate positively with TNF‐*α* and IL‐6 concentrations (Ammann et al. 2003). Observations of myocardial dysfunction improvement, tracked by echocardiography, occurred simultaneously with decreases in TNF‐*α*, IL‐10, IL‐8 and hs‐cTnI during the recovery process (Bouhemad et al. 2008). It is believed that cytokines, particularly IL‐10 and IL‐15, play a role in the development of myocardial injury in critically ill dogs with systemic inflammation (Langhorn, Oyama, et al. 2013). Moreover, to monitor and manage septicaemia caused by pyometra effectively, it is essential to examine the cytokines from immune system cells and also to keep track of any adverse effects they might have on the heart muscle (Haas, Kaup, and Neumann 2016). Therefore, understanding the relationship between cytokines and hs‐cTnI concentration is crucial (Diniz, Schwartz, and Collicchio‐Zuanaze 2007; Fransson et al. 2007; Karlsson et al. 2016). Studies on cytokines have been conducted to assess the post‐operative period in dogs with pyometra (Dąbrowski et al. 2015). However, no such studies exist for queens yet, so it is not possible to compare these findings with other studies.

Although none of the cats involved in our study had a documented history of heart disease, a limitation of our approach is that we did not definitively exclude the potential presence of undiagnosed heart conditions. In addition, we did not perform echocardiography measurements. Further understanding of the impact of pyometra on heart damage may be better informed through conducting more sensitive studies involving larger numbers of animals in the future.

## 5 Conclusion

To the best of our knowledge, this study is the first to explore the relationship between hs‐cTnI and Th1/Th2 cytokine polarization in queens afflicted with pyometra. A correlation was discovered between hs‐cTnI and CK concentrations and cytokines in queens suffering from pyometra. Understanding the cytokines involved in the immune response of queens with pyometra, and the changes in hs‐cTnI used for monitoring cardiac damage, is crucial for the development of treatment methods and preventative measures against this disease. Further research is required to enhance our knowledge about the relationship between hs‐cTnI and the functions of cytokines in the immune response in pyometra‐afflicted queens.

## Author Contributions

**Ta** **rik Safak**: writing–original draft, writing–review and editing, conceptualization, data curation, methodology, investigation. **Nevzat Saat**: project administration, investigation, funding acquisition, writing–review and editing. **Oznur Yilmaz‐Koc**: investigation, writing–review and editing. **Mert Turanli**: investigation, writing–review and editing. **Aslıhan Ayalp‐Erkan**: investigation, writing–review and editing. **Ali Risvanli**: investigation, writing–review and editing, supervision.

## Conflicts of Interest

The authors declare no conflicts of interest.

## Data Availability

The data that support the findings of this study are available from the corresponding author, upon reasonable request.

## References

[vms370125-bib-0001] Ammann, P. , M. Maggiorini , O. Bertel , et al. 2003. “Troponin as a Risk Factor for Mortality in Critically Ill Patients Without Acute Coronary Syndromes.” Journal of the American College of Cardiology 41, no. 11: 2004–2009. 10.1016/S0735-1097(03)00421-2. 12798573

[vms370125-bib-0002] Bartoskova, A. , P. Turanek‐Knotigova , J. Matiasovic , et al. 2012. “ *γ*δ T Lymphocytes Are Recruited Into the Inflamed Uterus of Bitches Suffering From Pyometra.” Veterinary Journal 194, no. 3: 303–308. 10.1016/j.tvjl.2012.05.024. 22771147

[vms370125-bib-0003] Bouhemad, B. , A. Nicolas‐Robin , C. Arbelot , M. Arthaud , F. Féger , and J. J. Rouby . 2008. “Isolated and Reversible Impairment of Ventricular Relaxation in Patients With Septic Shock.” Critical Care Medicine 36, no. 3: 766–774. 10.1097/CCM.0B013E31816596BC. 18431265

[vms370125-bib-0004] Bozza, F. A. , J. I. Salluh , A. M. Japiassu , et al. 2007. “Cytokine Profiles as Markers of Disease Severity in Sepsis: A Multiplex Analysis.” Critical Care 11: 1–8. 10.1186/cc5783 PMC2206478 17448250

[vms370125-bib-0005] Brady, C. A. , and C. M. Otto . 2001. “Systemic Inflammatory Response Syndrome, Sepsis, and Multiple Organ Dysfunction.” Veterinary Clinics of North America Small Animal Practice 31, no. 6: 1147–1162. 10.1016/s0195-5616(01)50097-2. 11727331

[vms370125-bib-0006] Dąbrowski, R. , K. Kostro , U. Lisiecka , M. Szczubiał , and L. Krakowski . 2009. “Usefulness of C‐Reactive Protein, Serum Amyloid A Component, and Haptoglobin Determinations in Bitches With Pyometra for Monitoring Early Post‐Ovariohysterectomy Complications.” Theriogenology 72, no. 4: 471–476. 10.1016/j.theriogenology.2009.03.017. 19477501

[vms370125-bib-0007] Dąbrowski, R. , J. Pastor , M. Szczubiał , et al. 2015. “Serum IL‐6 and IL‐10 Concentrations in Bitches With Pyometra Undergoing Ovariohysterectomy.” Acta Veterinaria Scandinavica 57: 1–4. 10.1186/s13028-015-0153-8. 26410584 PMC4584008

[vms370125-bib-0008] Dembic, Z. 2015. The Cytokines of the Immune System: The Role of Cytokines in Disease Related to Immune Response. New York, NY: Mica Haley.

[vms370125-bib-0009] Diniz, P. P. V. D. P. , D. S. Schwartz , and R. D. C. Collicchio‐Zuanaze . 2007. “Cardiac Trauma Confirmed by Cardiac Markers in Dogs: Two Case Reports.” Arquivo Brasileiro de Medicina Veterinária e Zootecnia 59: 85–89. 10.1590/S0102-09352007000100015.

[vms370125-bib-0010] dos Santos Silva, L. A. , L. G. C. Trautwein , M. M. T. Hidalgo , et al. 2024. “Renal Biomarkers, Clinical Parameters, and Renal Doppler Velocimetry in Bitches With Cystic Endometrial Hyperplasia—Pyometra Complex.” Reproduction in Domestic Animals 59, no. 1: e14518. 10.1111/rda.14518. 38268215

[vms370125-bib-0011] Fransson, B. A. , A. S. Lagerstedt , A. Bergstrom , et al. 2007. “C‐Reactive Protein, Tumor Necrosis Factor *α*, and Interleukin‐6 in Dogs With Pyometra and SIRS.” Journal of Veterinary Emergency and Critical Care 17, no. 4: 373–381. 10.1111/j.1476-4431.2006.00203.x.

[vms370125-bib-0012] Friolani, M. , A. M. Santana , F. A. Voorwald , et al. 2024. “Acute Phase Proteins, Hematological and Serum Biochemical Profiles of Female Dogs in Diestrus, Mucometra and Pyometra.” Arquivo Brasileiro de Medicina Veterinária e Zootecnia 76: 187–200. 10.1590/1678-4162-12921.

[vms370125-bib-0013] Haas, M. , F. J. Kaup , and S. Neumann . 2016. “Canine Pyometra: A Model for the Analysis of Serum CXCL8 in Inflammation.” Journal of Veterinary Medical Science 78, no. 3: 375–381. 10.1292/jvms.15-0415. 26522810 PMC4829503

[vms370125-bib-0014] Hagman, R. 2018. “Pyometra in Small Animals.” Veterinary Clinics of North America Small Animal Practice 48, no. 4: 639–661. 10.1016/j.cvsm.2018.03.001. 29933767

[vms370125-bib-0015] Hagman, R. , A. S. Lagerstedt , B. A. Fransson , A. Bergström , and J. Häggström . 2007. “Cardiac Troponin I Levels in Canine Pyometra.” Acta Veterinaria Scandinavica 49: 1–8. 10.1186/1751-0147-49-6. 17328800 PMC1839102

[vms370125-bib-0016] Hayati, F. , M. S. Ahrari‐Khafi , M. Hassankhani , M. Mansourian , and S. Asghari . 2016. “A Rare Radiographic Appearance of a Calcified Uterus in a Queen With Pyometra: A Case Report.” Veterinarni Medicina 61, no. 6: 357–360. 10.17221/167/2015-VETMED.

[vms370125-bib-0017] Heilmann, R. M. , and J. S. Suchodolski . 2015. “Is Inflammatory Bowel Disease in Dogs and Cats Associated With a Th1 or Th2 Polarization?” Veterinary Immunology and Immunopathology 168, no. 3–4: 131–134. 10.1016/j.vetimm.2015.10.008. 26672910

[vms370125-bib-0018] Hollinshead, F. , and N. Krekeler . 2016. “Pyometra in the Queen: To Spay or Not to Spay?” Journal of Feline Medicine and Surgery 18, no. 1: 21–33. 10.1177/1098612X15623114. 26733546 PMC11148877

[vms370125-bib-0019] Hori, Y. , M. Iguchi , Y. Heishima , et al. 2018. “Diagnostic Utility of Cardiac Troponin I in Cats With Hypertrophic Cardiomyopathy.” Journal of Veterinary Internal Medicine 32, no. 3: 922–929. 10.1111/jvim.15131. 29660794 PMC5980312

[vms370125-bib-0020] Jaffer, U. , R. G. Wade , and T. Gourlay . 2010. “Cytokines in the Systemic Inflammatory Response Syndrome: A Review.” HSR Proceedings in Intensive Care & Cardiovascular Anesthesia 2, no. 3: 161. 23441054 PMC3484588

[vms370125-bib-0021] Jursza, E. , M. P. Kowalewski , A. Boos , D. J. Skarzynski , P. Socha , and M. J. Siemieniuch . 2015. “The Role of Toll‐Like Receptors 2 and 4 in the Pathogenesis of Feline Pyometra.” Theriogenology 83, no. 4: 596–603. 10.1016/j.theriogenology.2014.10.023. 25481489

[vms370125-bib-0022] Jursza, E. , A. Z. Szóstek , M. P. Kowalewski , A. Boos , K. Okuda , and M. J. Siemieniuch . 2014. “LPS‐Challenged TNF*α* Production, Prostaglandin Secretion, and TNF*α*/TNFRs Expression in the Endometrium of Domestic Cats in Estrus or Diestrus, and in Cats With Pyometra or Receiving Medroxyprogesterone Acetate.” Mediators of Inflammation 2014: Article ID 689280. 10.1155/2014/689280. PMC4083830 25028529

[vms370125-bib-0023] Kakihana, Y. , T. Ito , M. Nakahara , K. Yamaguchi , and T. Yasuda . 2016. “Sepsis‐Induced Myocardial Dysfunction: Pathophysiology and Management.” Journal of Intensive Care 4: 1–10. 10.1186/s40560-016-0148-1. 27011791 PMC4804632

[vms370125-bib-0024] Karabulut, T. S. F. T. , and K. Sönmez . 2018. “Evaluation of Tissue Trauma and Healing on the Basis of Tumour Necrosis Factor—Alpha, Interleukin‐6 and C‐Reactive Protein in Peripheral Blood During and After Pyometra in Bitches.” Medycyna Weterynaryjna 74, no. 10: 658–664. 10.21521/mw.6122.

[vms370125-bib-0025] Karlsson, I. , R. Hagman , A. Johannisson , L. Wang , E. Karlstam , and S. Wernersson . 2012. “Cytokines as Immunological Markers for Systemic Inflammation in Dogs With Pyometra.” Reproduction in Domestic Animals 47: 337–341. 10.1111/rda.12034. 23279533

[vms370125-bib-0026] Karlsson, I. , R. Hagman , A. Johannisson , L. Wang , F. Södersten , and S. Wernersson . 2016. “Multiplex Cytokine Analyses in Dogs With Pyometra Suggest Involvement of KC‐Like Chemokine in Canine Bacterial Sepsis.” Veterinary Immunology and Immunopathology 170: 41–46. 10.1016/j.vetimm.2016.01.005. 26837616

[vms370125-bib-0027] Keskin, A. , G. Yilmazbas , R. Yilmaz , M. O. Ozyigit , and A. Gumen . 2009. “Pathological Abnormalities After Long‐Term Administration of Medroxyprogesterone Acetate in a Queen.” Journal of Feline Medicine and Surgery 11, no. 6: 518–521. 10.1016/j.jfms.2008.10.006. 19058986 PMC10832839

[vms370125-bib-0028] Kırbaş, A. , Ş. Değirmençay , A. Kilinc , and M. Eroğlu . 2021. “Evaluation of Serum Cardiac Troponin‐I Concentration and Cardiac Enzyme Activities in Neonatal Calves With Sepsis.” Israel Journal of Veterinary Medicine 76, no. 1: 4–11.

[vms370125-bib-0029] Labonté, J. , J. Dubuc , J. P. Roy , and S. Buczinski . 2018. “Prognostic Value of Cardiac Troponin I and L‐Lactate in Blood of Dairy Cows Affected by Downer Cow Syndrome.” Journal of Veterinary Internal Medicine 32, no. 1: 484–490. 10.1111/jvim.14874. 29205491 PMC5787165

[vms370125-bib-0030] Langhorn, R. , M. A. Oyama , L. G. King , et al. 2013. “Prognostic Importance of Myocardial Injury in Critically Ill Dogs With Systemic Inflammation.” Journal of Veterinary Internal Medicine 27, no. 4: 895–903. 10.1111/jvim.12105. 23678990

[vms370125-bib-0031] Langhorn, R. , and J. L. Willesen . 2016. “Cardiac Troponins in Dogs and Cats.” Journal of Veterinary Internal Medicine 30, no. 1: 36–50. 10.1111/jvim.13801. 26681537 PMC4913658

[vms370125-bib-0032] Langhorn, R. , J. L. Willesen , I. Tarnow , and M. Kjelgaard‐Hansen . 2013. “Evaluation of a High‐Sensitivity Assay for Measurement of Canine and Feline Serum Cardiac Troponin I.” Veterinary Clinical Pathology 42, no. 4: 490–498. 10.1111/vcp.12085. 24131244

[vms370125-bib-0033] Leonardi, F. , B. Passeri , A. Fussari , et al. 2008. “Cardiac Troponin I (cTnI) Concentration in an Ovine Model of Myocardial Ischemia.” Research in Veterinary Science 85: 141–144. 10.1016/j.rvsc.2007.09.010. 17961616

[vms370125-bib-0034] Maciel, G. S. , R. R. Uscategui , V. T. De Almeida , M. E. F. Oliveira , M. A. R. Feliciano , and W. R. R. Vicente . 2014. “Quantity of IL‐2, IL‐4, IL‐10, INF‐γ, TNF‐α and KC‐Like Cytokines in Serum of Bitches With Pyometra in Different Stages of Oestrous Cycle and Pregnancy.” Reproduction in Domestic Animals 49, no. 4: 701–704. 10.1111/rda.12360. 24975377

[vms370125-bib-0035] Maddens, B. , R. Heiene , P. Smets , et al. 2011. “Evaluation of Kidney Injury in Dogs With Pyometra Based on Proteinuria, Renal Histomorphology, and Urinary Biomarkers.” Journal of Veterinary Internal Medicine 25, no. 5: 1075–1083. 10.1111/j.1939-1676.2011.0772.x. 21848947

[vms370125-bib-0054] Otto, C. M. 2007. “Clinical Trials in Spontaneous Disease in Dogs: a New Paradigm for Investigations of Sepsis.” Journal of Veterinary Emergency and Critical Care 17, no. 4: 359–367. 10.1111/j.1476-4431.2007.00249.x.

[vms370125-bib-0036] Oyama, M. A. , and D. D. Sisson . 2004. “Cardiac Troponin‐I Concentration in Dogs With Cardiac Disease.” Journal of Veterinary Internal Medicine 18, no. 6: 831–839. 10.1111/j.1939-1676.2004.tb02629.x. 15638266

[vms370125-bib-0037] Pelander, L. , R. Hagman , and J. Häggström . 2008. “Concentrations of Cardiac Troponin I Before and After Ovariohysterectomy in 46 Female Dogs With Pyometra.” Acta Veterinaria Scandinavica 50: 1–8. 10.1186/1751-0147-50-35. 18786242 PMC2546406

[vms370125-bib-0038] Pugliese, M. , E. Napoli , R. La Maestra , et al. 2023. “Cardiac Troponin I and Electrocardiographic Evaluation in Hospitalized Cats With Systemic Inflammatory Response Syndrome.” Veterinary Sciences 10, no. 9: 570. 10.3390/vetsci10090570. 37756092 PMC10538112

[vms370125-bib-0039] Safak, T. , A. Risvanli , and Z. Asci‐Toraman . 2022. “Th1/Th2 Cytokine Polarization in Milk According to Different Pathogens Causing Subclinical Mastitis in Cows.” Mljekarstvo 72, no. 2: 105–113. 10.15567/mljekarstvo.2022.0204.

[vms370125-bib-0040] Sant'Anna, M. C. , L. G. P. Giordano , K. K. M. C. Flaiban , E. E. Muller , and M. I. M. Martins . 2014. “Prognostic Markers of Canine Pyometra.” Arquivo Brasileiro de Medicina Veterinária e Zootecnia 66: 1711–1717. 10.1590/1678-6859.

[vms370125-bib-0053] Satilmis, F. 2023. Pyometra in Queens ‐ Changes in Haemato‐Biochemical Parameters. Acta Scientiae Veterinariae. 10.22456/1679-9216.126420.

[vms370125-bib-0041] Schober, P. , C. Boer , and L. A. Schwarte . 2018. “Correlation Coefficients: Appropriate Use and Interpretation.” Anesthesia & Analgesia 126, no. 5: 1763–1768. 10.1213/ANE.0000000000002864. 29481436

[vms370125-bib-0042] Singh, L. K. , M. K. Patra , G. K. Mishra , et al. 2020. “Prospects of Diagnostic and Prognostic Biomarkers of Pyometra in Canine.” Asian Pacific Journal of Reproduction 9, no. 4: 166–173. 10.4103/2305-0500.288584.

[vms370125-bib-0043] Termelioğlu, L. , H. Kalender , and S. Erat . 2022. “Comparison of Vaginal Flora, Vaginal Cytology, Blood Values and Hormone Level of Cats in Different Reproductive Periods.” International Journal of Veterinary and Animal Research 5, no. 1: 10–18.

[vms370125-bib-0044] Tümer, K. Ç. , M. Çalişkan , and T. Şafak . 2021. “Serum Cardiac Troponin I Concentrations in Ewes Diagnosed With Parturient Paresis: Correlation With Blood Ionized Calcium and Conventional Cardiac Enzymes.” Large Animal Review 27: 143–147.

[vms370125-bib-0045] Tümer, K. Ç. , H. Özdemir , and H. Eröksüz . 2020. “Evaluation of Cardiac Troponin I in Serum and Myocardium of Rabbits With Experimentally Induced Polymicrobial Sepsis.” Experimental Animals 69, no. 1: 54–61. 10.1538/expanim.19-0046. 31462610 PMC7004812

[vms370125-bib-0046] Tümer, K. C. , and T. Safak . 2022. “Serum Cardiac Troponin I Concentration Increases in Sheep With Uterine Torsion.” Small Ruminant Research 216: 106784. 10.1016/j.smallrumres.2022.106784.

[vms370125-bib-0047] Vijay, A. , M. Shafiuzama , S. Hemalatha , and M. Gokulakrishnan . 2021. “Haemato‐Biochemical Changes in Pyometra Affected Bitches During and After Ovariohysterectomy.” Pharma Innovation Journal 10, no. 4: 04–08.

[vms370125-bib-0048] Wesselowski, S. , J. Lidbury , A. B. Saunders , S. G. Gordon , J. S. Suchodolski , and J. M. Steiner . 2023. “Analytical Validation, Sample Stability, and Clinical Evaluation of a New High‐Sensitivity Cardiac Troponin I Immunoassay for Use in Dogs, With Comparison to a Previous Ultrasensitive Assay.” PLoS ONE 18, no. 7: e0288801. 10.1371/journal.pone.0288801. 37463140 PMC10353792

[vms370125-bib-0049] Wu, A. H. , and A. S. Jaffe . 2008. “The Clinical Need for High‐Sensitivity Cardiac Troponin Assays for Acute Coronary Syndromes and the Role for Serial Testing.” American Heart Journal 155, no. 2: 208–214. 10.1016/j.ahj.2007.10.016. 18215588

[vms370125-bib-0050] Xavier, R. G. C. , P. H. S. da Silva , H. D. Trindade , et al. 2022. “Characterization of *Escherichia coli* in Dogs With Pyometra and the Influence of Diet on the Intestinal Colonization of Extraintestinal Pathogenic *E. coli* (ExPEC).” Veterinary Sciences 9, no. 5: 245. 10.3390/vetsci9050245. 35622773 PMC9144190

[vms370125-bib-0051] Zambelli, D. , and F. Prati . 2006. “Ultrasonography for Pregnancy Diagnosis and Evaluation in Queens.” Theriogenology 66, no. 1: 135–144. 10.1016/j.theriogenology.2006.04.004. 16716385

[vms370125-bib-0052] Zhu, J. , and W. E. Paul . 2008. “CD4 T Cells: Fates, Functions, and Faults.” Blood 112: 1557–1569. 10.1182/blood-2008-05-078154. 18725574 PMC2518872

